# Cultural Adaptation of the Actionable Health App Evaluation in Japan: Protocol for a Web-Based Modified Delphi Expert Consensus Study

**DOI:** 10.2196/44469

**Published:** 2023-11-03

**Authors:** Kengo Yokomitsu, Hikari N Takashina, Yoshitake Takebayashi, Seiji Muranaka

**Affiliations:** 1 School of Psychological Sciences University of Human Environments Ehime Japan; 2 Research Center for Child Mental Development Chiba University Chiba Japan; 3 School of Medicine Fukushima Medical University Fukushima Japan; 4 Graduate School of Human Sciences Osaka University Osaka Japan

**Keywords:** mobile, eHealth, mHealth apps, mobile health, modified Delphi study, Japan

## Abstract

**Background:**

With an increase in both the number of mental health disorders people are experiencing and the difficulty in accessing mental health care, the demand for accessible mental health care services has increased. The use of mobile devices has allowed people to receive care in their daily lives without restrictions on time or location. However, the majority of publicly available mobile health apps are not evidence-based, and the top-rated apps are not always safe or user-friendly and may not offer clinically beneficial results.

**Objective:**

This study aims to create a cultural adaptation of the American Psychiatric Association’s comprehensive app evaluation framework in Japan using a web-based modified Delphi expert consensus.

**Methods:**

A web-based modified Delphi study includes developing the Japanese version of the comprehensive app evaluation framework and 3 Delphi rounds. In the first round, our working group sends a questionnaire to the panelists, who then complete it. In the second and third rounds, the working group sends a questionnaire and a summary of the panelists’ answers based on each of the previous rounds. The panelists answer the questionnaires based on this summary. The summarization procedure is automated to help reduce the biases that can be generated when panelists’ answers are summarized and when the panelists receive them. The working group sends only the result of the summarization with the next round’s questionnaire. All interactions between the working group and the panelists will be conducted on Qualtrics (Qualtrics Japan LLC), a questionnaire platform. To culturally validate the comprehensive mental health app evaluation framework, participants from the following three categories will be recruited in Japan: (1) researchers, (2) practitioners, and (3) app developers.

**Results:**

This study received funding from a crowdfunding campaign in Japan (April 2023). The Delphi study began in January 2023 and will be completed in December 2023. We had already completed the translation of the 105 original app evaluation item questions by December 2022.

**Conclusions:**

While the need for treatment using mental health apps is increasing, no framework that can be used to develop a centralized database for health apps is available or accessible, and no consensus has been reached among stakeholders in Japan about an appropriate framework. The results of the web-based modified Delphi method presented in this paper may provide direction for the development and use of mental health apps in the future among the relevant stakeholders. Furthermore, this study will enhance recognition of the framework among researchers, clinicians, mental health app developers, and users, in addition to devising new instruments to help users or practitioners efficiently choose the right app for their situations.

**International Registered Report Identifier (IRRID):**

PRR1-10.2196/44469

## Introduction

Problems with mental health, such as mental health disorders, affect people internationally. According to the Global Burden of Diseases, Injuries, and Risk Factors Study, the 2 most disabling mental disorders, depression and anxiety disorders, ranked among the top 25 leading causes of burden worldwide in 2019 [1]. In addition, the COVID-19 pandemic has had an enormous impact on mental health. For example, the World Health Organization [2] indicated that there was a remarkable increase in mental health problems in the general population; loneliness and a positive COVID-19 diagnosis increased the risk of suicidal thoughts. The COVID-19 pandemic led to a 27.6% increase in cases of major depressive disorders and a 25.6% increase in cases of anxiety disorders in 2020 [3]. Furthermore, COVID-19 has made it more difficult for people facing mental health problems to access outpatient mental health services [2].

With the rise of mental health problems and the increasing difficulty in access to in-person mental health care services, the demand for accessible mental health care services has significantly increased. The use of mobile devices, which are now owned by a majority of people, has made it possible for individuals to access care in their daily lives without being restricted by place and time [4-6]. As of 2017, more than 318,000 health apps existed worldwide; this is twice as many as were available in 2015. Of 318,000 health apps, 490 apps targeted mental health and behavioral disorders [7]. In Japan, a systematic search of the Google Play Store and Apple App Store between June 4 and June 11, 2021, found that 172 mental health apps are available [8]. Moreover, a previous study of Japanese university students found that the mobile health (mHealth) apps usage rate was 32.1% [9]. Considering that 36% of adults who owned smartphones or tablets in a national sample of the United States [10] had mHealth apps on their devices, the usage rate of mental health apps in Japan is estimated to be about the same as in other countries.

Recent reviews have found that mHealth apps could be effective in reducing symptoms of depression and anxiety in adults and youth [11,12]. Unfortunately, some of the apps available are unreliable or harmful. Most publicly available mHealth apps are not evidence-based, and top-rated apps on the app store are not always safe or user-friendly and may not offer clinically beneficial results [13,14]. Similar problems have been reported in Japan [15].

It is difficult for service users (eg, clinical therapists, patients, the general public, and those without mental health concerns) to ensure that apps are safe, evidence-based, usable, and offer clinically beneficial results [16]. Multiple scales and frameworks have been developed to evaluate mHealth apps [17,18]. These scales and frameworks include scales for evaluating the quality, safety, usability, and importance of apps [19]. However, the majority of scales have been developed for app developers to evaluate their own apps [19], and more inclusive tool apps are necessary to help users select the right app based on their conditions and preferences.

The App Evaluation Model framework proposed by the American Psychiatric Association (APA) is useful for addressing this issue [20]. This framework was constructed via a 6-step process that involved harmonizing the 961 app evaluation questions included in 45 existing app evaluation frameworks, removing duplicate, redundant, and nonrelevant questions, and then grouping the remaining 357 questions into 5 priority levels: background information, privacy and security, evidence-based, ease of use, and data integration [21]. Even then, there was no centralized database where users can glance at how various health apps perform when assessed via the framework. Therefore, Lagan et al [16] translated APA’s framework for evaluating apps into a set of objective questions that can be published on the internet; the questions from the APA model were operationalized into the 105 objective questions that are either binary or numeric. The 105 objective questions are aligned with the levels of the APA framework, with the 5 levels arranged in a pyramid format to reinforce the need to first consider access, safety, and privacy. There were some additions and alterations to several questions to reflect ongoing feedback from stakeholders after a 2-day summit in December 2019 [16]. The main difference between the user version of the Mobile App Rating Scale, which is the most used mHealth apps evaluation scale, and the app evaluation model is that the app evaluation model does not score questions or produce summary scores about the quality of app safety, usability, etc, but instead allows the user to judge what is important and a good match for them.

Currently, no framework that can be used to develop a centralized database for health apps is available and accessible, and no consensus has been reached among experts and clinicians in Japan about an appropriate framework. The aim of this paper is to describe the study protocol that will be used to validate the adaptation of the 105 objective questions in Japan and develop a Japanese version of the framework among stakeholders involved in mental health apps. In the absence of empirical evidence to reach such a consensus, various methods can be used to synthesize opinions based on expertise and scientific background. In this study, we will use the Delphi method, which ensures anonymity and allows opinions to be expressed without group pressure. The Delphi method has been used to formulate various guidelines and models [22,23]. Our Delphi study is conducted to examine whether the 105 objective questions are comprehensive, content-appropriate, and relevant to this domain as criteria for evaluating mental health apps. The target audience for this framework includes people involved in the research, clinical practice, and app development of health apps throughout Japan. The reason for targeting these people whose native language is Japanese is also to identify difficulties in understanding items that were not noticed during the Japanese translation process.

## Methods

### Ethical Considerations

Participation in this study is voluntary. The emailed Delphi survey invitation includes the following information for potential panelists: presentation of the researcher responsible for the study, description of the study, reasons for the selection of the expert, procedures to be followed to participate, estimation of the time required, expectations regarding the expert (including the importance of participating in all rounds of the consultation), the promise of anonymity and participation recognition, and rewards for participation (payment of 20,000 JPY [approximately US $143] at the end of the study). Before participation in this study, informed consent was obtained from all panelists.

### Design

This research procedure is planned with reference to the Conducting and Reporting Delphi Studies (CREDES) guidelines [24] and reviews of the Delphi study [25,26]. The classic Delphi techniques have the following characteristics [27]: (1) a working group organizes the Delphi study; (2) the working group recruits a panelist’s group of individuals with some expertise on the topic; (3) the group compiles a questionnaire with a list of statements that the experts rate for agreement; (4) the group collects responses from the panelists using the questionnaire; (5) the group gives anonymous feedback to each panelist about how their responses compare to the rest of the panelists; (6) each panelist is able to revise their responses to the questionnaire after receiving the feedback; and (7) responses converge across rounds of questionnaires, with some statistical criterion being used to define consensus. Since developing a Japanese version of the comprehensive app evaluation framework serves as a starting point for the modified Delphi technique, we refer to our Delphi study as a modified Delphi study. The expected duration of the study is 12 months, which began in January 2023. To culturally validate the comprehensive mental health app framework in Japan, Japanese participants will be recruited from three categories: (1) researchers, (2) practitioners, and (3) app developers.

### Procedure of the Web-Based Modified Delphi Study

Table 1 shows the procedure of developing the Japanese version of the comprehensive app evaluation framework. The Delphi method includes 3 rounds, with evaluation panelists answering anonymously in each round. In each round of the Delphi survey, the working group will pilot the round with 1 researcher, 1 practitioner, and 1 app developer prior to initiation. The individuals who participate in the pilot will not participate in the Delphi process. In the first round, our working group will send a questionnaire to the panelists, who will complete it within 4 weeks. If any panelist does not complete the evaluation within 4 weeks and does not respond to communications from the working group, the panelist would be considered to have dropped out of the study and will be excluded from the analysis. In the second and third rounds, the working group will send a questionnaire and a summary of the panelists’ answers based on each previous round. The panelists answer the questionnaires based on this summary. The summarization procedure is automated to reduce biases that may occur when summarizing panelists’ answers and providing those summaries to the panelists. The working group sends the result of the summarization with the next round’s questionnaire. Each round’s summary includes the following: the quantitative result for each item’s question and explanation, the qualitative comments and explanation for each evaluation, and suggestions for modifying (revising, removing, and adding) items. When the panelist suggests revision or addition of items, the working group members will review the proposals within 3 weeks after the panelists’ response. The working group would discuss the items and judge whether each revised or added item’s expression is adequate. An independent liaison from the working group will make contact with the panelists, and the information about the panelists will be masked to the working group members who are participating in the discussion. These discussions will be conducted by 2 or more members.

**Table 1:** Steps of the study process.

		Working group	Panelist
**Preparation**			
	Step 1 (completed)	Developing the app evaluation items Japanese version	N/A^a^
	Step 2 (completed)	Defining the inclusion and exclusion criteria for panelists	N/A
	Step 3	Sampling the Delphi round’s panelists	Determine whether or not to participate in this study
	Step 4	Developing the Delphi questionnaire	N/A
**Delphi round**			
	First round	Conducting the pilot study Analysis	Evaluating the appropriateness of items in the final version of the 105 objective questions and suggesting notations for each item
	Second round	Conducting the pilot study Providing feedback to panelists on the summary of the first round Analysis	Judging the appropriateness of items in the final version of the 105 objective questions and suggesting notations for each item
	Final round	Conducting the pilot study Providing feedback on the summary of the second round Final analysis and publication	Judging the appropriateness of items in the final version of the 105 objective questions and suggesting notations for each item

^a^N/A: not applicable.

### Develop Questionnaires

The 105 original evaluation items were translated in accordance with the guidelines provided by the International Society for Pharmacoeconomics and Outcomes Research task force [28]. First, forward translation from English to Japanese was performed independently by 2 of the authors (KY and HNT). Then, a professional English translator who was English-Japanese bilingual and blinded to the 105 objective questions translated the provisional app evaluation item back into English. The 2 English versions of the app evaluation item questions (ie, the original and back-translated versions) were reconciled by KY, HNT, and John Torous (original 105 objective questions’ author), and only minor discrepancies were found. These discrepancies were discussed until a consensus was reached. The original author (John Torous) evaluated the finalized English version of the app evaluation item questions and confirmed that the original meanings of the items, instructions, and responses had been maintained throughout the translation procedure.

### Administer Questionnaires

Panelists will complete evaluations in a 2-stage structure (the 105 items and the 11 key levels). The 105 objective questions are constructed by the following key levels: app origin, functionality, app store attributes, accessibility, privacy and security, inputs, outputs, clinical foundation, features, engagement style, and interoperability and data sharing. At the beginning of each key level, the questionnaire explains the key level, and panelists will be asked to assess the appropriateness of the item that comprises the key level. At that time, we will ask panelists to suggest additional items if needed. After the evaluation of key level, panelists will assess 105 objective questions and descriptions. Panelists will be asked to respond on a 9-point scale (1=not at all appropriate to 9=quite appropriate) regarding whether the item is appropriate to evaluate apps. When the panelists assign a score of 1-5, they will be asked to provide free-text feedback that includes suggestions for changing the wording.

### Synthesize Answers

In keeping with the described methods, satisfactory agreement for each round will require both (1) all responses >5 (ie, no participant disagreement) and (2) 60% (n=31) participation in voting in each round. These criteria were conservatively set with reference to several previous studies [29,30]. In addition, satisfactory consensus for each round will require the following: the smaller the IQR, the greater the consensus; an IQR of 0 or 1 indicates a very strong consensus, while an IQR of 2 indicates a strong consensus [31]. IQR includes the difference between the 75th percentile (third quartile) and the 25th percentile (first quartile). The items that meet these criteria will be removed for the next round of the Delphi. In addition, for free-text feedbacks, 2 or more people of the working group would discuss the panelists’ responses regarding the addition or modification of items, whether to adopt the responses in the next round, and in case of adoption, how the items should be added or modified. If no conclusion is reached through discussion, the working group members who were not able to participate on the discussion day will be asked for their opinions. For this reason, the discussions will be recorded on video and via the minutes of the meeting.

### Elaborate Selection Criteria for Evaluation Panelists

#### Overview

The panelists will consist of 51 researchers, practitioners, and app developers. Inclusion and exclusion criteria of the panelists in each category are as follows.

Researchers who have experience contributing to at least 1 study for psychological assessment or treatment with mHealth apps will be included. The number of relevant publications, the manner in which they contribute to the research, and the degree of contribution are not required; however, if they are involved in assessment, treatment, development, usability or feasibility studies, protocol studies, reviews, preprints, conference papers, etc, they will be considered candidates for panelists.

Any practitioner who has experience with at least 1 app that assists in the treatment of procedure to a patient or client who is facing mental health issues will be considered a candidate. Practitioners include psychiatrists and clinical psychologists. Clinical experience related to mental health care is not required. Given the state of app development in Japan, we anticipate that the panelists would be limited, which would undermine their inclusiveness. Therefore, we include those who have used an app for patients for treatment or assessment purposes, regardless of the mental health app.

For app developers, candidates must have experience developing at least 1 app related to mental health care. There is no minimum requirement for years of experience in mental health app development that must be met. However, panelists who participated in the development of mental health app items will be excluded.

#### Make a List of Panelists

Panelists will be recruited based on the inclusion and exclusion criteria that will be considered in the primary list. Subsequently, snowball sampling will be conducted to expand the pool of panelists.

#### Dissemination

The preponderant involvement of various stakeholders throughout the study will offer the possibility of disseminating the results and progress of this study during the conduction of this study. Following that, diverse activities will take place to transfer knowledge. For example, scientific papers will be published, and conferences will be held to share the results with researchers. These activities could include working with the media to produce commentary papers, features, and stakeholder interviews on research findings. Workshops will be organized with professionals. A podcast will also be available for people interested in delving deeper into the results and related topics and listening to interviews and discussions with experts. Public lectures for citizens are also planned to disseminate the results of the study and explain how to use the app evaluation items to the general public. These activities will be conducted both on-site and on the internet, and records of the activities will be made available through the website and social networking sites developed for the study.

## Results

We have already completed the translation of the 105 original app evaluation item questions in December 2022. We compiled a list of panelists willing to participate in the Delphi study. We anticipated the first Delphi round to commence in January 2023, and that all Delphi rounds will be completed by December 2023. Finally, the results of each Delphi round will be anonymously reported, and the report will be distributed among all panelists who participated in the Delphi study. This report will include an indication of the distribution of panelists’ ratings, including their comments and suggestions. The results will be presented both quantitatively and qualitatively. We expect this study to be published in a peer-reviewed journal and presented at national and international conferences.

## Discussion

### Principal Considerations

While the need for treatment using mental health apps and the development of mental health apps to meet this need are increasing [32], no framework which can be used to develop a centralized database for health app is available and accessible, and no consensus has been reached among stakeholders in Japan about an appropriate framework. The web-based modified Delphi method presented in this paper aims to reach a consensus on an app framework, and thus ensure the direction of use and development of mental health apps in the future.

We expect that this web-based modified Delphi method will incorporate many different stakeholders involved in mental health apps (researchers, clinicians, and mental health app developers), facilitate the application of the comprehensive app evaluation framework in Japan, and provide valuable insights that will contribute to future developments in this field.

Furthermore, we expect that this work will raise awareness of the framework among some stakeholders involved in mental health apps, in addition to leading to the development of tools that will enable users or practitioners to efficiently choose the right app for their situations.

### Strengths and Limitations

The majority of studies aiming at cultural adaptation of a survey have thus far failed to follow the CREDES guidance. This study is the second CREDES-based cultural adaptation of a scale in Japan. Furthermore, the panelist group in this Delphi study includes clinical experts, researchers, and developers to use diverse perspectives. This limitation will be due to the diversity of panelists, as there will be only 51 panelists in total, which is 17 participants for each domain. The CREDES prefers 50 participants for Delphi studies; therefore, the planned number of participants is acceptable. On the other hand, since this study plans to conduct the first round for 51 panelists at the time of study participation, it is possible that the number of panelists will decrease over time.

### Conclusions

This study will develop the Japanese version of the comprehensive app evaluation framework that has reached a consensus among some stakeholders involved in the mental health app. It will generate useful insights for people involved in research, clinical practice, and app development regarding mental health apps throughout Japan and suggest the direction of use and development of mental health apps in the future. Furthermore, this study will enhance recognition of the framework among researchers, clinicians, mental health app developers, and users, in addition to developing new instruments to help users or practitioners efficiently choose the right app for their situations.

## Abbreviations

APA: American Psychiatric Association
CREDES: Conducting and Reporting Delphi Studies
mHealth: mobile health

## Data Availability

Data sharing is not applicable to this paper as no data sets were generated or analyzed during this study.
